# Supplementary material for: Realistic AI-generated climate disaster images decrease support for climate action when artificial origin is suspected
Source: Commun Sustain. 2026 May 20;1(1):84. doi: 10.1038/s44458-026-00092-0 (PMC13190298; doi:10.1038/s44458-026-00092-0)
Supplement: Supplementary file 2 — Supplementary Information [file 44458_2026_92_MOESM2_ESM.pdf]

Supplementary Web Appendix

1. Sociodemographic Information 2

2. Experimental Stimuli 3

3. Image Generation & Prompts 6

4. Pretest Results 10

5. Measures 11

6. Correlations 13

7. Confirmatory Factor Analyses 16

8. Predictor coding for SEM 19

9. ANOVA Results 22

10. Additional Analyses 23

Supplementary References 25

1. Sociodemographic Information

Table A.1 Sociodemographic characteristics of the samples

Characteristic	Study 1 Sample (*N* = 534)	Study 2 Sample (*N* = 552)	Study 3 Sample (*N* = 1,494)
Age	*M* = 38.7 years	*M* = 44.7	*M* = 41.2 years
Gender (male)	48.3 %	44.7 %	51.1 %
Ethnicity[a]			
White	70 %	82.2 %	76.3 %
Black or African American	26 %	11.6 %	18.6 %
American Indian or Alaska Native	1.3 %	2.0 %	1.9 %
Asian	5.2 %	4.5 %	4.4 %
Native Hawaiian or Pacific Islander	0.4 %		0.1 %
Hispanic	5.2 %	0.2 %	4.6 %
Education			
Did not high school	0.4 %	0.4 %	0.4 %
High school graduate	26.6 %	28.6 %	24.0 %
Associate's degree	10.5 %	13.8 %	10.0 %
Bachelor's degree	39.7 %	38.9 %	39.9 %
Advanced degree (Masters, Professional, Ph.D.)	22.8 %	18.3 %	25.7 %
Political Orientation			
Very conservative	11.2 %	6.9 %	11.6 %
Conservative	25.7 %	19.2 %	28.0 %
Middle of the road	24.3 %	23.0 %	23.7 %
Liberal	26.6 %	32.8 %	24.8 %
Very liberal	12.2 %	18.1 %	11.9 %
Prior Disaster Experience[b]			
Yes	69.9 %	35.9 %	
No	30.1 %	61.8 %	
Don't know	-	2.3 %	

Note. [a] Percentages for ethnicity may sum to more than 100% because participants could select multiple categories. [b] Because the stimulus contexts differed across studies, the wording of this item varied slightly. In Study 1, participants were asked, "Have there been any floods in your local area in the past year?" In Study 2, the question was broadened to include multiple disaster types: "Have there been any wildfires, hurricanes, and/or floods in your local area in the past year?".

2. Experimental Stimuli

Study 1

1) Text-only control condition

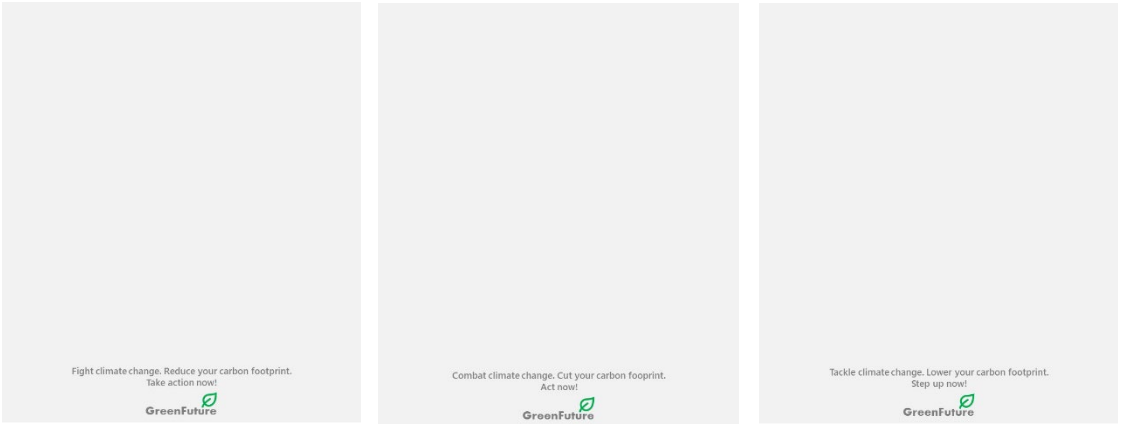

2) Low visual realism condition

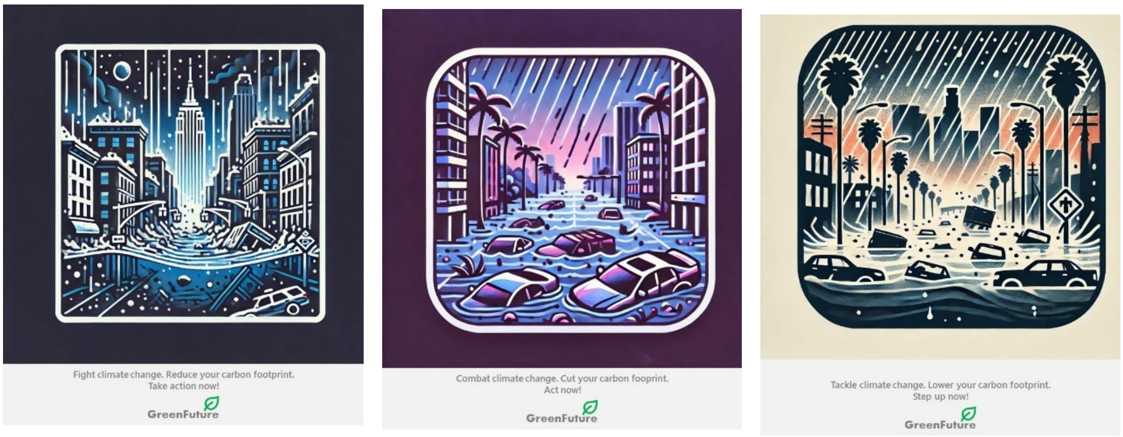

3) High visual realism condition

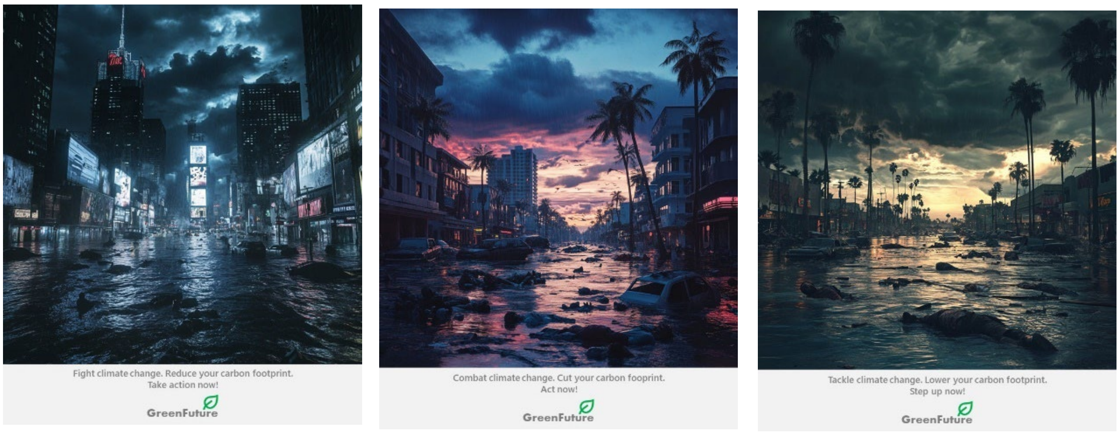

Figure A.1 Experimental stimuli used in Study 1.

Study 2

1) Text-only control condition

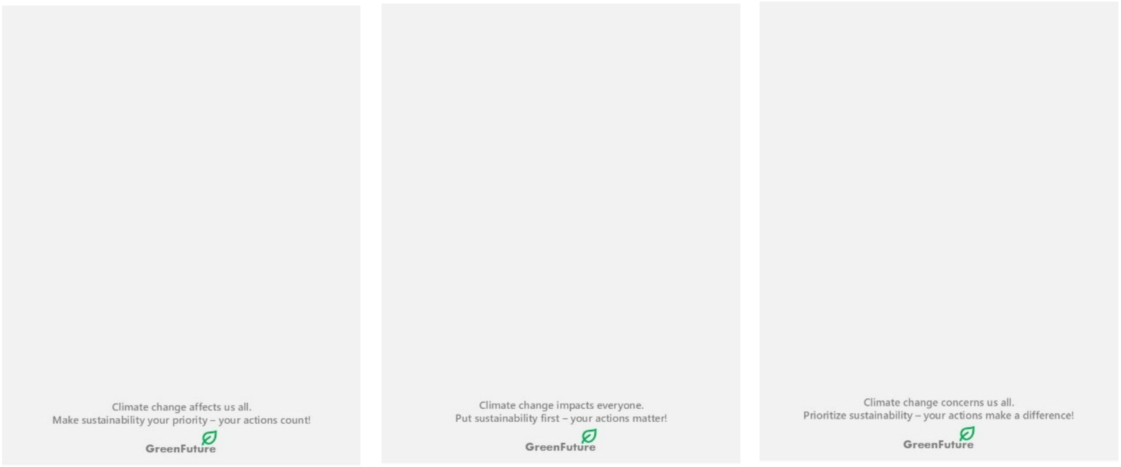

2) Low visual realism condition

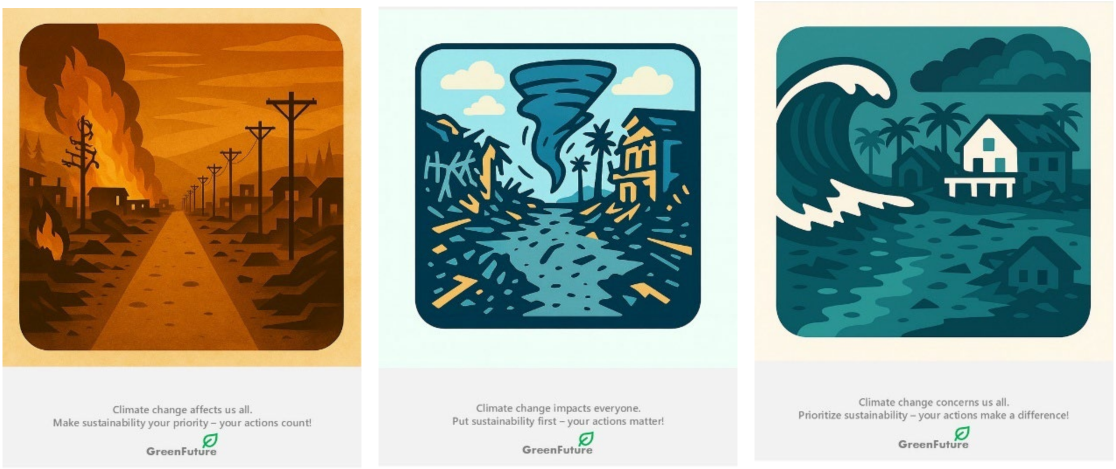

3) High visual realism condition

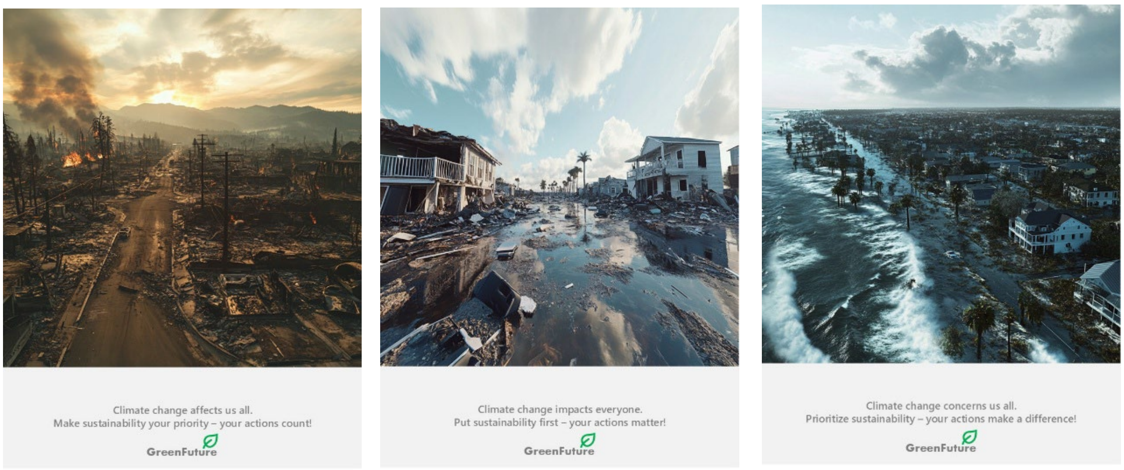

Figure A.2 Experimental stimuli used in Study 2.

Study 3

1) Text-only control condition

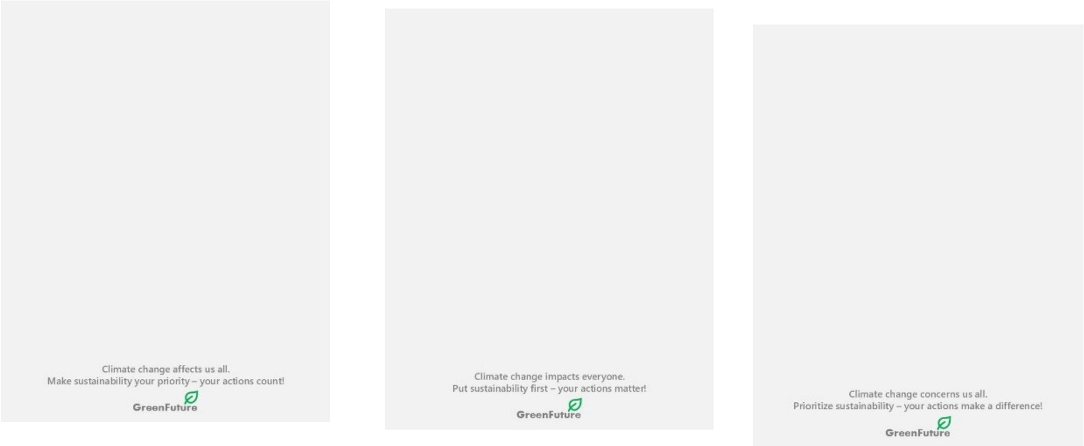

2) High visual realism condition

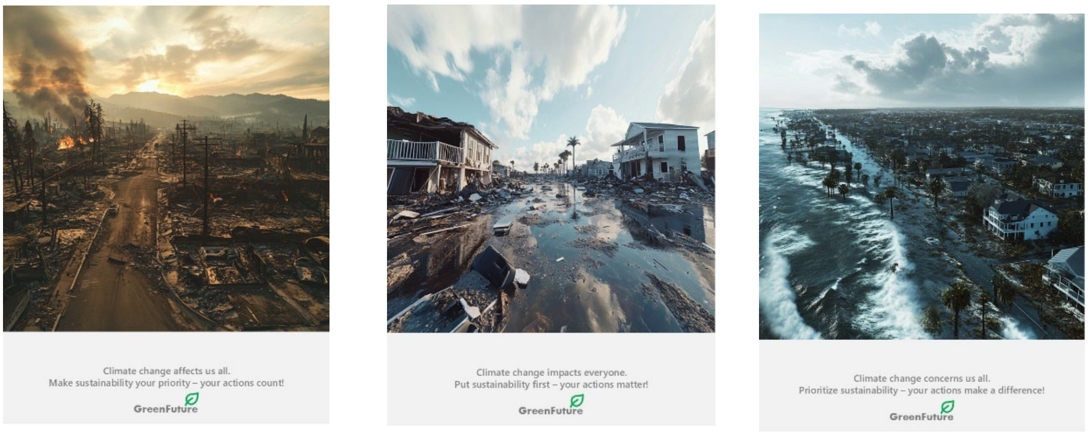

3) High visual realism + AI origin label condition

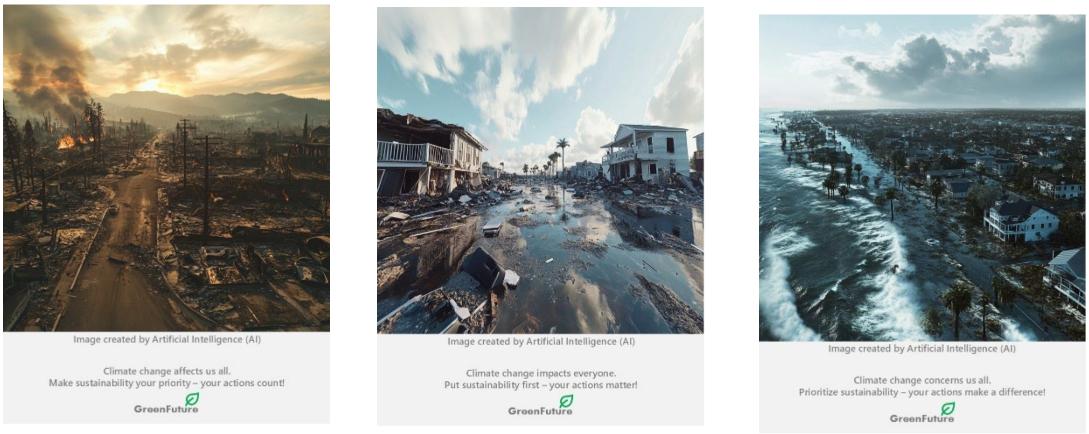

Figure A.3 Experimental stimuli used in Study 3.

3. Image Generation & Prompts

Study 1

Table A.2 Prompts used in Study 1

High visual realism images		
An apocalyptic scene of New York Broadway underwater, destroyed, with torrential rainfall, a stormy and dark sky, in 8K resolution	An apocalyptic scene of Miami Sunset Boulevard underwater, destroyed, with torrential rainfall, a stormy and dark sky, in 8K resolution	An apocalyptic scene of Los Angeles Sunset Boulevard underwater, destroyed, with torrential rainfall, a stormy and dark sky, in 8K resolution

Low visual realism images		
Use this image and create a schematic, abstract icon of New York Broadway underwater, destroyed, with torrential rainfall. Show a street-level view and use the same color palette as the attached image. The icon should be square-shaped.	Use this image and create a schematic, abstract icon of Miami Sunset Boulevard underwater, destroyed, with torrential rainfall. Show a street-level view and use the same color palette as the attached image. The icon should be square-shaped.	Use this image and create a schematic, abstract icon of Los Angeles Sunset Boulevard underwater, destroyed, with torrential rainfall. Show a street-level view and use the same color palette as the attached image. The icon should be square-shaped.

Prompt refinements. Initial prompts were iteratively refined to more clearly operationalize differences in visual realism while holding other visual properties constant.

For the high-realism condition, prompts were iteratively revised to optimize output realism. Early prompt versions included highly detailed descriptions, but excessive detail often led to visual artifacts (e.g., incomplete object rendering or structural inconsistencies). Refining prompts toward more concise formulations while preserving key scene elements resulted in more coherent and visually realistic outputs.

For the high-realism condition, prompts were refined by adding cues associated with high visual fidelity (e.g., "8K resolution," environmental descriptors such as lighting and weather conditions) to elicit more photorealistic renderings. Such cues are known to increase perceived realism in generative image models (see van Berlo et al., 2024).

To ensure comparability across conditions, prompts were further adapted the low-visual realism prompts to explicitly specify a street-level perspective. This adjustment was necessary because, while high-realism images were typically generated with a street-level viewpoint by default, low-realism image generation tended to produce more abstract, bird's-eye perspectives. Explicitly controlling for viewpoint ensured that differences between conditions reflected variation in visual realism rather than unintended differences in perspective.

Prompts for low-visual realism images were additionally refined to enforce a consistent color palette across conditions, thereby controlling for color-based differences and isolating the effect of visual realism.

Finally, prompts for low-realism images were modified to specify a square format to standardize composition and reinforce the icon-like, graphic nature of the images. Without this constraint, generated icons varied in shape and occupied different proportions of the visual field, resulting in unintended variation in size and composition across stimuli.

Stimulus selection and post-processing. Multiple candidate images were generated for each prompt and condition until no substantial variation in outputs was observed (i.e., saturation). From this pool, images were jointly evaluated and selected in consultation with communication scholars at our research institute to identify those that most clearly represented the intended stimuli. The selected images were subsequently validated in a

pretest to ensure that they adequately captured the intended differences in visual realism.

No post-processing was applied. All images were used exactly as generated by the respective generative AI tool.

Study 2

Table A.3 Prompts used in Study 2

High visual realism images		
A city in California being destroyed by wildfires, street-level view, 8K resolution	A city in Louisiana being destroyed by a hurricane, street-level view, 8K resolution	A city in Florida being destroyed by a coastal flood wave, street-level view, 8K resolution

Low visual realism images		
Use this image and create a schematic, abstract icon of a city in California being destroyed by wildfires. Show a street-level view and use the same color palette as the attached image. The icon should be square-shaped.	Use this image and create a schematic, abstract icon of a city in Louisiana being destroyed by a hurricane. Show a street-level view and use the same color palette as the attached image. The icon should be square-shaped.	Use this image and create a schematic, abstract icon of a city in Florida being destroyed by a coastal flood wave. Show a street-level view and use the same color palette as the attached image. The icon should be square-shaped.

Prompt refinements. For the high-realism condition, further simplification of prompts improved output realism. Reducing prompts to essential scene elements enhanced visual coherence and minimized artifacts associated with overly complex prompt specifications.

Stimulus selection and post-processing. The same procedure as in Study 1 was applied. No post-processing was performed. All images were used exactly as generated by the respective generative AI tool.

4. Pretest Results

Table A.4 Repeated-measures ANOVA results for stimulus pretests (by image set)

	Pretest Study 1 N = 35						Pretest Study 2 N = 35					
Outcome	Image Set 1		Image Set 2		Image Set 3		Image Set 1		Image Set 2		Image Set 3	
Realism	Low	High	Low	High	Low	High	Low	High	Low	High	Low	High
Perceived Colorfulness	3.4 (1.7)	3.9 (1.8)	4.9 (1.7)	4.6 (1.6)	3.7 (1.4)	3.6 (1.8)	3.6 (1.5)	3.8 (1.4)	4.8 (1.5)	4.3 (1.3)	3.3 (1.5)	3.3 (1.4)
Perceived Visual realism	2.4[a] (1.5)	5.0[a] (1.8)	2.3[a] (1.5)	4.6[a] (1.9)	2.3[a] (1.4)	5.0[a] (1.7)	2.1[a] (1.4)	5.7[a] (1.3)	2.0[a] (1.4)	6.3[a] (0.8)	1.8[a] (1.0)	5.4[a] (1.6)

Note. Means are presented above, and standard deviations are presented below in brackets.
In both pretests, participants rated all images. Colorfulness was measured with a single item on a 7-point scale ranging from 1 (*not colorful*) to 7 (*extremely colorful*) (Pretest Study 1: $M = 4.2$, $SD = 1.4$; Pretest Study 2: $M = 3.8$, $SD = 1.0$) [1]. Visual realism was assessed with three items ("This is a photographic image," "This is a naturalistic image," "This is a realistic image") using a 7-point Likert-type scale from 1 (*not at all*) to 7 (*very much*) (Pretest Study 1: $M = 3.6$, $SD = 1.2$; Pretest Study 2: $M = 3.9$, $SD = 0.7$). Items were averaged to create a visual realism composite score (Pretest Study 1: $\alpha = .93$; Pretest Study 2: $\alpha = .82$). For each image set, means with superscripts differ at $p < .05$.

5. Measures

Table A.5 Overview of experimental measures, items, and descriptive statistics

Type	Measure	Items	Study 1			Study 2			Study 3		
			Reliability	*Mean*	*SD*	Reliability	*Mean*	*SD*	Reliability	*Mean*	*SD*
Message perceptions[a]	Perceived Threat to Freedom	Indicate how much you agree with the following statements: The message tried to make a decision for me. The message tried to manipulate me. The message tried to pressure me.	α = .89	3.07	1.79	α = .92	2.62	1.69	α = .89	2.97	1.73
Sender perceptions[b]	Perceived Trustworthiness	What was your perception of the sender of the message? Dishonest – Honest Unreliable – Reliable Insincere – Sincere Untrustworthy – Trustworthy							α = .97	4.81	1.98
Emotions[c]		How did the message make you feel?									
	Anger	Angry Annoyed	α = .81	2.28	1.62	α = .69	1.95	1.33	α = .82	2.11	1.54
	Fear	Fearful Scared	α = .95	2.85	1.85	α = .96	2.75	1.86	α = .94	2.53	1.79
	Sadness	Sad Depressed	α = .81	2.75	1.72	α = .83	2.77	1.78	α = .83	2.60	1.73
	Despair	Desperate Demoralized	α = .81	2.23	1.47	α = .79	1.99	1.36	α = .81	1.97	1.36
	Guilt	Guilty	α = .90	2.15	1.50	α = .91	2.04	1.45	α = .87	2.12	1.47

		Ashamed									
Cognitions	Counter-arguing[d]	Indicate your agreement with the following statements. While looking at the message, I had _________ thoughts about it.<br>Critical<br>Negative	$\alpha = .65$	3.56	1.70	$\alpha = .77$	3.0	1.76	$\alpha = .65$	3.22	1.74
Persuasion	Willingness to make personal sacrifices for climate action[e]	Indicate your agreement with the following statements<br>I am willing to pay more for climate-friendly products.<br>I am willing to adhere to the law as well as other climate regulations, even if they restrict my daily life.<br>Higher prices for climate-friendly energy are acceptable.<br>It is important for me to buy climate-friendly food, even if it is more expensive.<br>I am willing to sacrifice some everyday convenience for climate protection.	$\alpha = .93$	4.41	1.68	$\alpha = .93$	4.36	1.62	$\alpha = .93$	4.62	1.65

Note. All items were measured using a 7-point Likert scale. [a] adopted from Dillard & Shen[2]
[b] adopted from Ohanian[3]
[c] adopted from Dillard & Peck[4]
[d] adopted from Bünzli et al.[5]
[e] adopted from Bilandzic et al.[6]

6. Correlations

Study 1

Table A.6 Correlation matrix of measures used in Study 1

	Perc Threat Freedom	Emot Anger	Emot Fear	Emot Sad	Emot Despair	Emot Guilt	Counter-arguing	Willing Make Pers Sacrifices
Perc.Threat Freedom	1	.595**	.092*	.086*	.213**	.131**	.502**	-.330**
Emot Anger	.595**	1	.279**	.292**	.416**	.284**	.568**	-.200**
Emot Fear	.092*	.279**	1	.814**	.680**	.611**	.163**	.319**
Emot Sad	.086*	.292**	.814**	1	.751**	.613**	.157**	.300**
Emot Despair	.213**	.416**	.680**	.751**	1	.697**	.243**	.240**
Emot Guilt	.131**	.284**	.611**	.613**	.697**	1	.224**	.260**
Counter-arguing	.502**	.568**	.163**	.157**	.243**	.224**	1	-.169**
Willing Make Pers Sacrifices	-.330**	-.200**	.319**	.300**	.240**	.260**	-.169**	1

Note. Values represent Pearson correlation coefficients. N = 534. *p ≤ .05. **p ≤ .01. ***p ≤ .001.

Study 2

Table A.7 Correlation matrix of measures used in Study 2

	Perc Threat Freedom	Emot Anger	Emot Fear	Emot Sad	Emot Despair	Emot Guilt	Counter-arguing	Willing Make Pers Sacrifices
Perc.Threat Freedom	1	.477***	-.096*	-.081	.039	-.003	.442***	-.419***
Emot Anger	.477***	1	.266***	.245***	.381***	.313***	.508***	-.194***
Emot Fear	-.096*	.266***	1	.824***	.665***	.644***	.084*	.353***
Emot Sad	-.081	.245***	.824***	1	.743***	.667***	.095*	.348***
Emot Despair	.039	.381***	.665***	.743***	1	.675***	.191***	.252***
Emot Guilt	-.003	.313***	.644***	.667***	.675***	1	.167***	.316***
Counter-arguing	.442***	.508***	.084*	.095*	.191***	.167***	1	-.144***
Willing Make Pers Sacrifices	-.419***	-.194***	.353***	.348***	.252***	.316***	-.144***	1

Note. Values represent Pearson correlation coefficients. N = 552. *p ≤ .05. **p ≤ .01. ***p ≤ .001.

Study 3

Table A.8 Correlation matrix of measures used in Study 3

	Perc.Threat Freedom	Perc Trust	Emot Anger	Emot Fear	Emot Sad	Emot Despair	Emot Guilt	Counter-arguing	Willing Make Pers Sacrifices
Perc.Threat Freedom	1	-.374**	.548**	.064*	.078**	.227**	.113**	.479**	-.388**
Perc Trust	-.374**	1	-.319**	.131**	.107**	.036	.135**	-.234**	.455**
Emot Anger	.548**	-.319**	1	.308**	.328**	.434**	.304**	.547**	-.249**
Emot Fear	.064*	.131**	.308**	1	.790**	.662**	.627**	.255**	.204**
Emot Sad	.078**	.107**	.328**	.790**	1	.715**	.617**	.235**	.163**
Emot Despair	.227**	.036	.434**	.662**	.715**	1	.681**	.324**	.144**
Emot Guilt	.113**	.135**	.304**	.627**	.617**	.681**	1	.257**	.193**
Counter-arguing	.479**	-.234**	.547**	.255**	.235**	.324**	.257**	1	-.169**
Willing Make Pers Sacrifices	-.388**	.455**	-.249**	.204**	.163**	.144**	.193**	-.169**	1

Note. Values represent Pearson correlation coefficients. N = 1,494. *p ≤ .05. **p ≤ .01. ***p ≤ .001.

7. Confirmatory Factor Analyses

Study 1

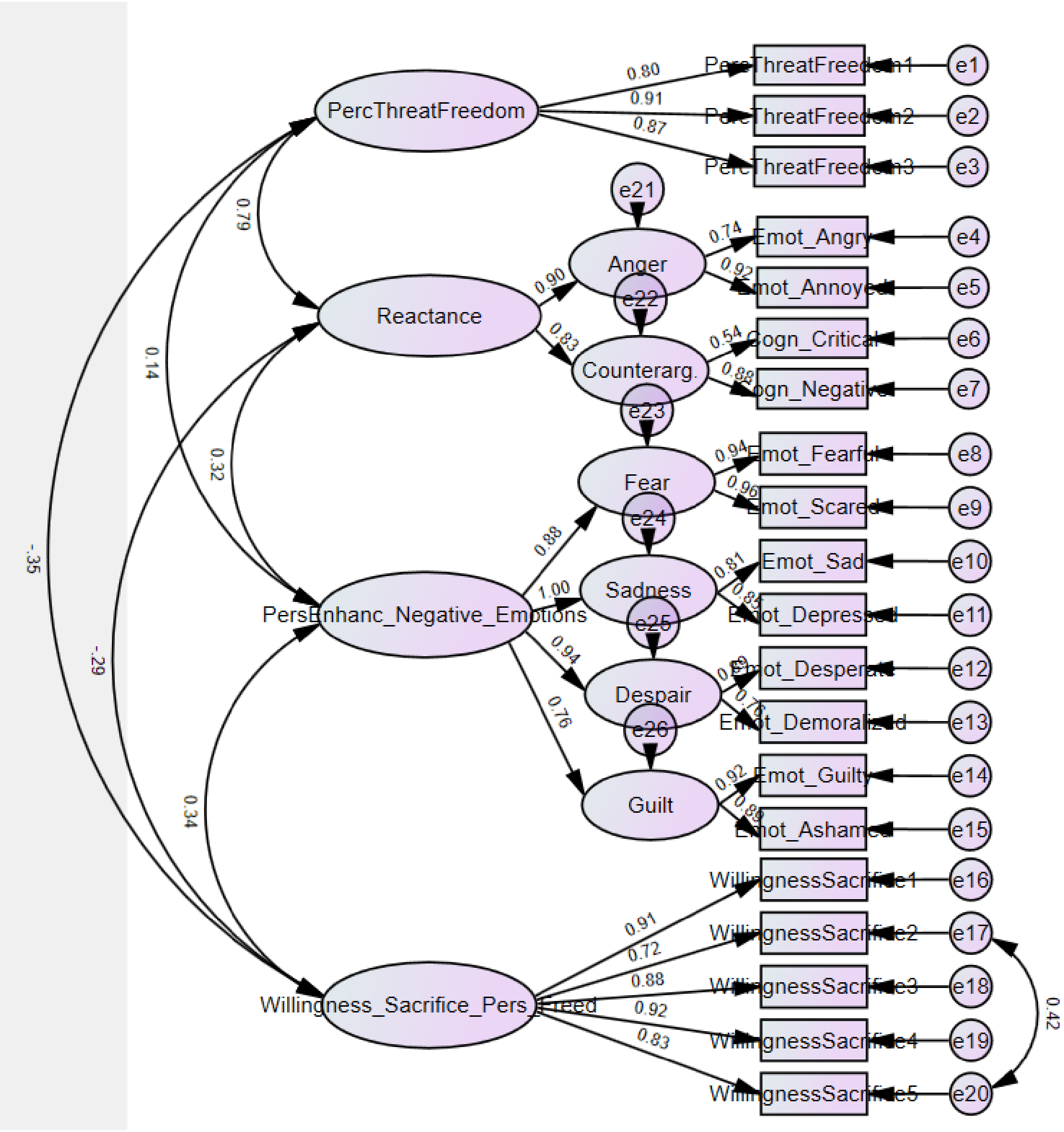

Figure A.4 Measurement model for Study 1 with standardized factor loadings from the confirmatory factor analysis.

Note. Initial model fit was acceptable, $\chi^2(158, N = 534) = 672.437$, $p < .001$; RMSEA = .078 (90% confidence interval [CI] = .072–.084); CFI = .939; and TLI = .927. However, modification indices suggested that model fit could be improved by allowing two items of the *willingness to sacrifice personal freedom* factor to covary. After making this modification, model fit improved to $\chi^2(157, N = 534) = 589.558$, $p < .001$; RMSEA = .072 (90% confidence interval [CI] = .066–.078); CFI = .949; and TLI = .938.
The model demonstrated sufficient convergent and discriminant validity.

Study 2

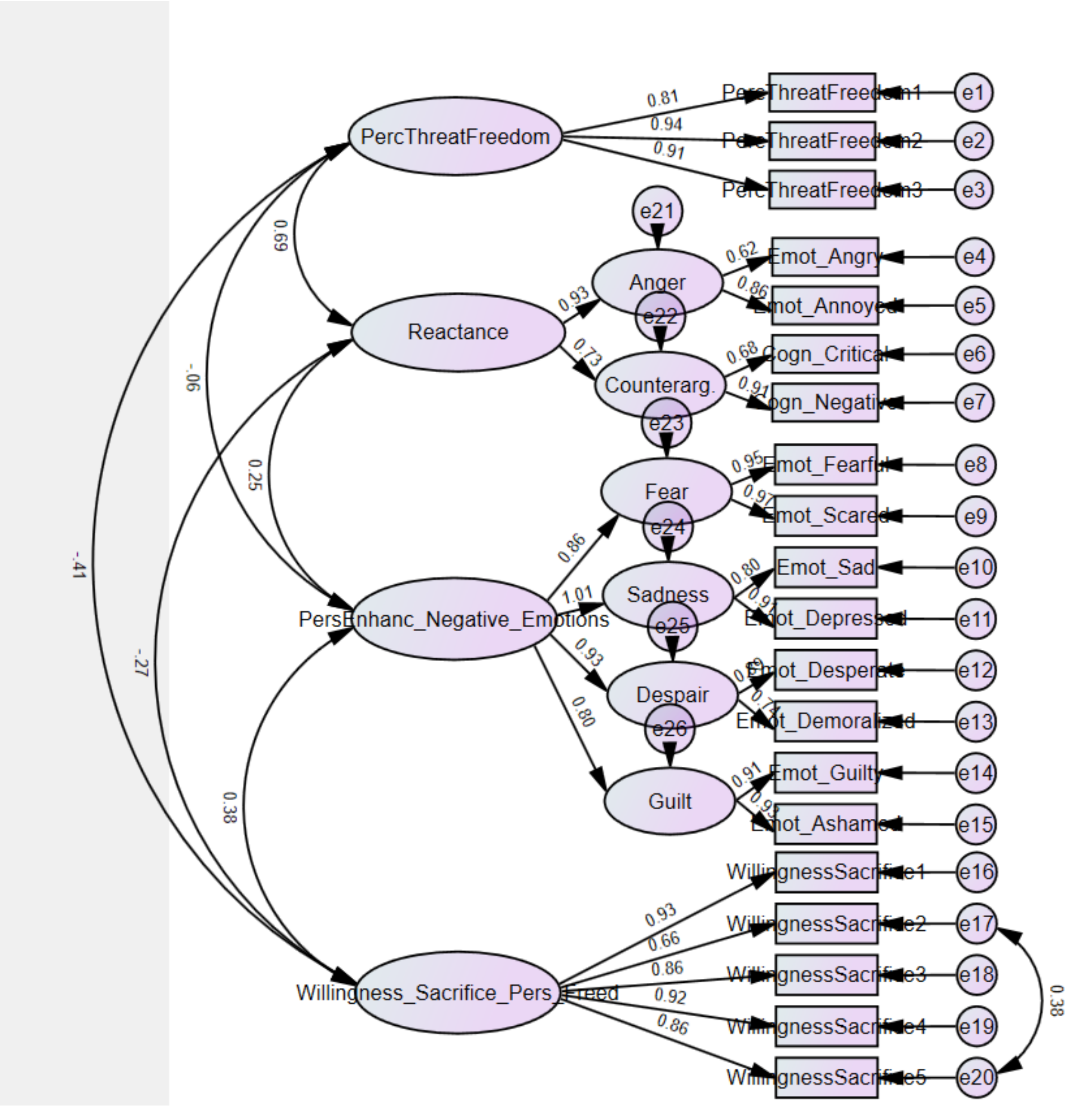

Figure A.5 Measurement model for Study 2 with standardized factor loadings from the confirmatory factor analysis.

Note. Initial model fit was acceptable, $\chi^2(158, N = 552) = 744.147$, $p < .001$; RMSEA = .082 (90% confidence interval [CI] = .076–.088); CFI = .937; and TLI = .924. However, modification indices suggested that model fit could be improved by allowing two items of the *willingness to sacrifice personal freedom* factor to covary. After making this modification, model fit improved to $\chi^2(157, N = 552) = 674.882$, $p < .001$; RMSEA = .077 (90% confidence interval [CI] = .071–.083); CFI = .944; and TLI = .932.
The model demonstrated sufficient convergent and discriminant validity.

Study 3

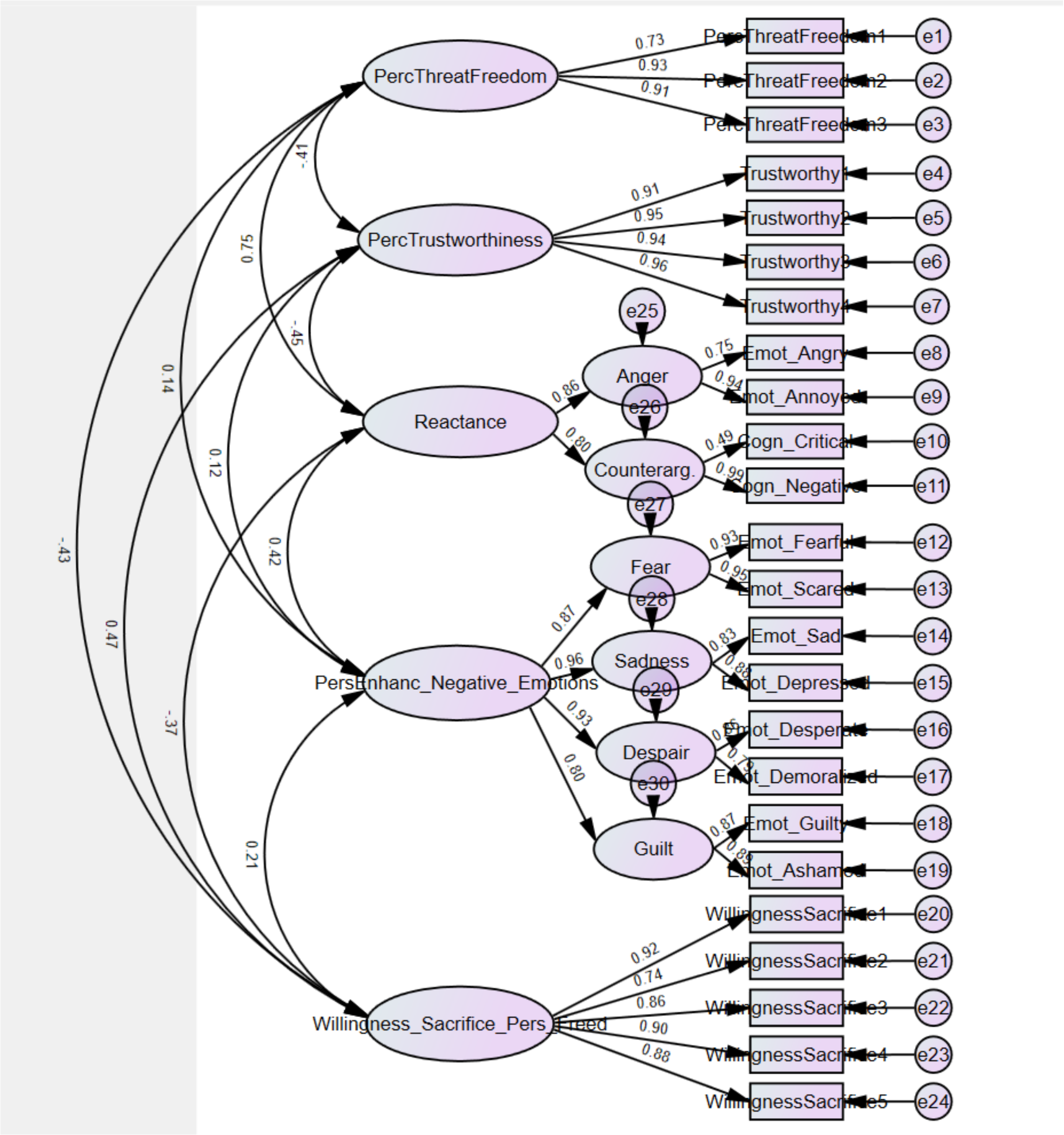

Figure A.6 Measurement model for Study 3 with standardized factor loadings from the confirmatory factor analysis.
Note. Model fit was good, χ^2(236, N = 1,494) = 1387.972, p < .001; RMSEA = .057 (90% confidence interval [CI] = .054–.060); CFI = .964; and TLI = .958. The model demonstrated sufficient convergent and discriminant validity.

8. Predictor coding for SEM

Study 1

Table A.9 Predictor coding

Image Variation	Variable Coding	
	**Low-realism AI image present**	**High-realism AI image present**
Text only	0	0
Low visual realism	1	0
High visual realism	0	1

Study 2

Table A.10 Predictor coding

**Image Variation**	**Variable Coding**	
	**Low-realism AI image present**	**High-realism AI image present**
Text only	0	0
Low visual realism	1	0
High visual realism	0	1

Study 3

Table A.11 Predictor coding

Image Variation	Variable Coding<br>Presence of AI image: perceived as photograph	Presence of AI image: perceived as AI-generated or unsure	Presence of AI image + AI cue
Text only	0	0	0
High visual realism (perceived as real photo)	1	0	0
High visual realism (perceived as AI-generated or unsure)	0	1	0
High visual realism + AI origin label	0	0	1

9. ANOVA Results

Table A.12 ANOVA (analysis of variance) results showing the influence of AI-generated climate disaster imagery on individuals' willingness to make personal sacrifices for climate action

Outcome	Study 1 N = 534			Study 2 N = 552			Study 3 N = 1,494			
	Text-only (N = 177)	Low visual realism (N = 175)	High visual realism (N = 182)	Text-only (N = 174)	Low visual realism (N = 189)	High visual realism (N = 189)	Text-only (N = 514)	High visual realism		High visual realism + AI origin label (N = 473)
								Image perceived as real photo (N = 148)	Image perceived as AI-generated or unsure (N = 359)	
Willingness to make personal sacrifices	*M* = 4.2 [a] *SD* = 1.6	*M* = 4.6 [a] *SD* = 1.6	*M* = 4.5 *SD* = 1.7	*M* = 4.6 *SD* = 1.7	*M* = 4.2 *SD* = 1.6	*M* = 4.3 *SD* = 1.6	*M* = 4.8 [a] *SD* = 1.6	*M* = 4.9 [b] *SD* = 1.6	*M* = 4.3 [ab] *SD* = 1.8	*M* = 4.6 [(a*)] *SD* = 1.6

Note. Within each study, means sharing a superscript are significantly different at ($p < .05$) or for (*) at $p < .10$.

10. Additional Analyses

In this section, we present findings on the extent to which exposure to AI-generated climate impact imagery influences individuals' perceived threat of climate change. Perceived threat was measured using six items adapted from Bilandzic et al.[6] and rated on a 7-point Likert scale ranging from 1 (*strongly disagree*) to 7 (*strongly agree*): *"Climate change affects my own life", "Climate change affects everyone", "Climate change causes high costs", "In the future, climate change will affect humans' lives significantly", "Climate change also has a lot of advantages"* (reverse coded), "*Humans will cope well with the challenges of climate change"* (reverse coded) (Study 1: $M = 5.2$, $SD = 1.2$, $\alpha = .82$; *Study 2:* $M = 5.2$, $SD = 1.2$, $\alpha = .80$).

For Study 1, an analysis of variance (ANOVA) examining climate threat perceptions across conditions yielded, $F(2, 531) = 2.450$, $p = .087$, $\eta^2_p=.009$, which is above the conventional significance threshold ($p = .05$).

As shown in Table A.13, climate threat perceptions in Study 2 and Study 3 did not differ significantly across conditions, Study 2: $F(2, 549) = .988$, $p = .373$, $\eta^2_p=.004$; Study 3: $F(3, 1490) = .544$, $p = .652$, $\eta^2_p=.001$. Overall, the findings indicate consistently high levels of perceived climate threat across all conditions, suggesting that participants already possess a heightened awareness of climate change risks. This pattern may reflect a ceiling effect, whereby threat perceptions are already near the upper limit of the measurement scale, leaving little room for further increases in response to climate disaster imagery.

Table A.13 The effects of AI-generated climate disaster images on perceived threat of climate change

	Study 1<br>N = 534			Study 2<br>N = 552			Study 3<br>N = 1,494			
Outcome	Text-only<br>(N = 177)	Low visual realism<br>(N = 175)	High visual realism<br>(N = 182)	Text-only<br>(N = 174)	Low visual realism<br>(N = 189)	High visual realism<br>(N = 189)	Text-only<br>(N = 514)	High visual realism		High visual realism + AI origin label<br>(N = 473)
								Image perceived as real photo<br>(N = 148)	Image perceived as AI-generated or unsure<br>(N = 359)	
Perceived threat of climate change	*M* = 5.0<br>*SD* = 1.3	*M* = 5.3<br>*SD* = 1.2	*M* = 5.2<br>*SD* = 1.3	*M* = 5.5<br>*SD* = 1.2	*M* = 5.3<br>*SD* = 1.5	*M* = 5.4<br>*SD* = 1.3	*M* = 5.2<br>*SD* = 1.2	*M* = 5.2<br>*SD* = 1.2	*M* = 5.2<br>*SD* = 1.4	*M* = 5.3<br>*SD* = 1.2

Note. Within each study, means sharing a superscript are significantly different at ($p < .05$) or for (*) at $p < .10$.

Supplementary References

1. Dubey, R., Hardy, M. D., Griffiths, T. L. & Bhui, R. AI-generated visuals of car-free US cities help improve support for sustainable policies. *Nat. Sustain.* **7**, 399–403 (2024).
2. Dillard, J. P. & Shen, L. On the nature of reactance and its role in persuasive health communication. *Commun. Monogr.* **72**, 144–168 (2005).
3. Ohanian, R. Construction and validation of a scale to measure celebrity endorsers' perceived expertise, trustworthiness, and attractiveness. *J. Advert.* **19**, 39–52 (1990).
4. Dillard, J. P. & Peck, E. Affect and persuasion. Emotional responses to public service announcements. *Communic. Res.* **27**, 461–495 (2000).
5. Bünzli, F., Dillard, J. P., Li, Y. & Eppler, M. J. When visual communication backfires: Reactance to three aspects of imagery. *Communic. Res.* **52**, 683–713 (2025).
6. Bilandzic, H., Kalch, A. & Soentgen, J. Effects of goal framing and emotions on perceived threat and willingness to sacrifice for climate change. *Sci. Commun.* **39**, 466–491 (2017).
